# The determinants of immunization coverage among children aged between 12 and 35 months: a nationwide cross-sectional study in Lao People’s Democratic Republic

**DOI:** 10.1186/s12889-022-14522-w

**Published:** 2022-12-03

**Authors:** Yasunori Ichimura, Naoki Yanagisawa, Moe Moe Thandar, Chansay Pathammavong, Kongxay Phounphenghuk, Phonethipsavanh Nouanthong, Chankham Tengbriacheu, Bouaphane Khamphaphongphane, Lauren Elizabeth Franzel-Sassanpour, Tae Un Yang, Hendrikus Raaijmakers, Tomomi Ota, Kenichi Komada, Masahiko Hachiya, Shinsuke Miyano

**Affiliations:** 1 Bureau of International Health Cooperation, National Center for Global Health and Medicine, 1-21-1, Toyama, Shinjuku, Tokyo, 162-8655 Japan; 2 National Immunization Program, Ministry of Health, Simuang Road, Vientiane, Lao People’s Democratic Republic; 3 Institute Pasteur du Laos, National Immunization Technical Advisory Group, Ministry of Health, Samsenthai Road, Ban Kao-Gnot, Sisattanak district, Vientiane, Lao People’s Democratic Republic; 4 Mother and Child Health Center, Ministry of Health, Simuang Road, Vientiane, Lao People’s Democratic Republic; 5 National Center Laboratory and Epidemiology, KM3 Thadeua road, Sisattanak District, Vientiane, Lao People’s Democratic Republic; 6 Vaccine-Preventable Diseases and Immunization section, World Health Organization Representative Office in the Lao People’s Democratic Republic, 125 Saphanthong Road, Unit 5, Ban Saphangthongtai, Sisattanak District, Vientiane, Lao People’s Democratic Republic; 7 Health and Nutrition section, United Nations Children’s Fund Lao People’s Democratic Republic, KM3 Thadeua road, Sisattanak District, Vientiane, Lao People’s Democratic Republic

**Keywords:** Full immunization, Immunization coverage, Extended program on immunization (EPI), Cross-sectional study, Lao People’s Democratic Republic

## Abstract

**Background:**

Immunization is one of the most important public health interventions for reducing morbidity and mortality in children. However, factors contributing to low immunization coverage are not fully understood in the Lao People’s Democratic Republic (Lao PDR). Therefore, this study aimed to identify factors associated with full immunization coverage among children between 12 and 35 months, providing up-to-date information for immunization programs in Lao PDR.

**Methods:**

We analyzed the subpopulation of a nationwide cross-sectional survey using a multistage cluster sampling procedure to evaluate the measles and rubella seroepidemiology. In addition, we categorized children aged between 12 and 35 months into two groups: “fully immunized” children with a birth dose of Bacillus Calmette and Guérin vaccine, hepatitis B vaccine (Hep B), one and three doses for the measles-containing vaccine (MCV) and pentavalent vaccine and pneumococcal conjugate vaccine (PCV) and “partially immunized” children who missed any dose of vaccine. Immunization coverage was calculated as the ratio of “fully immunized” to the total. We compared the groups’ demographic characteristics and health service utilization as independent variables. Multivariate logistic regression was used to assess the relationship between immunization coverage, various demographic factors, and health service utilization.

**Results:**

Overall, 256 of the 416 targeted pairs were included in the analysis. In total, 67.6% of the children were fully immunized. Childbirth at hospitals or health facilities (adjusted odds ratio: 9.75, 95% confidence interval: 5.72–16.62, *p* < 0.001) was the predictor of full immunization coverage. The 83 children in the partially immunized groups were attributed to Hep B at birth (46, 55.4%), three doses of PCV (34, 41.0%), and the first dose of the MCV (27, 32.5%).

**Conclusion:**

Our study elucidated that the immunization status among children aged between 12 and 35 months in Lao PDR is satisfactory in improving access to healthcare by strengthening communication with residents regarding health service utilization, and expanding mobile outreach services may play a pivotal role in this endeavor. Further research is warranted to evaluate efforts to increase immunization coverage and target populations with limited access to healthcare.

## Background

It is important to prevent vaccine-preventable diseases through immunization and maintain and improve the population’s health. Immunization protects individuals and reduces the number of such infections in society by improving collective immunity when many people are immunized. The Expanded Program on Immunization (EPI) was initiated in 1974 by the World Health Organization (WHO) and the United Nations Children’s Fund (UNICEF) to promote vaccination to protect children worldwide against preventable infectious diseases [1]. Initially, tuberculosis, polio, diphtheria, tetanus, whooping cough, and measles were selected for vaccination. Subsequently, with new vaccines added, EPI works with other public health programs to improve the health of all people everywhere [1].

In the Lao People’s Democratic Republic (PDR), the national immunization program launched the measles vaccine in 1982/1984 [2–5]. However, measles is still endemic due to immunization difficulties and surveillance in remote mountainous areas where most people live. Therefore, a pilot measles campaign targeting children aged 9–59 months was conducted in two provinces in 2000, with the remainder of the country covered in 2001 to improve measles control [2, 6]. In Lao PDR, supplementary immunization activities (SIAs) for measles were performed in 2007 and 2011 [6]. The rubella vaccine was introduced in the 2011 SIA and subsequently included in the National Immunization Program (NIP) in 2012 [6]. Lao PDR introduced a routine second dose of the measles-containing vaccine in the NIP in 2017. In 2019, a subnational immunization campaign was conducted for children aged between 6 months and 10 years [6]. Between 2000 and 2020, hepatitis B, Haemophilus influenza type B, polio (inactivated polio vaccine), pneumococcal bacteria, rotavirus, Japanese encephalitis virus, and human papillomavirus vaccines were introduced into the NIP [6]. The first national multistage random cluster sampling survey to identify the sociodemographic factors affecting immunization was conducted in 2014. Following the survey, the national EPI conducted two SIAs specifically on measles. The Eighth National Health Sector Development Plan 2016–2020 aims for at least 90% immunization coverage by 2020. Interventions to increase routine immunization coverage and to improve access to vaccines have been implemented in Lao PDR, for example, by developing cold chains to build the capacity of healthcare workers to deliver vaccines [7]. Conversely, the measles and hepatitis B vaccines (Hep B) administered at birth in 2019 were still low, at approximately 70% [8]. Therefore, there is a need to conduct a national survey to evaluate the interventions implemented recently and broaden the understanding of the factors influencing child immunization.

## Methods

### Aim and design of the study

**Fig. 1:**
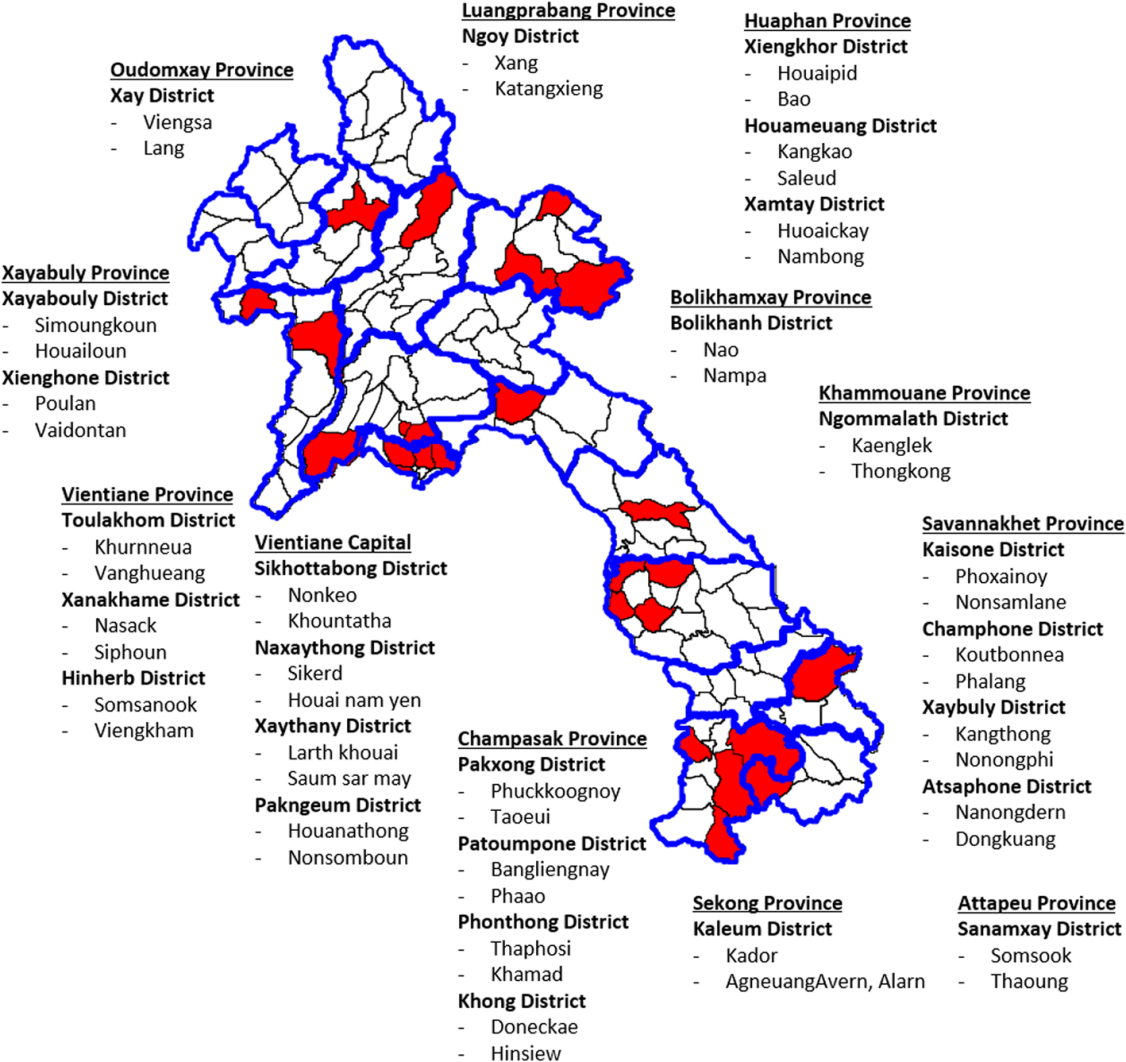
The map of selected 52 villages. This figure shows the selected 52 villages on the stratified multistage cluster method. Twenty-six districts were randomly selected as primary sampling units from all districts in Lao PDR using probability proportional to size (PPS), and two villages were selected as secondary sampling units from each district using PPS (in total, 52 villages)

This analysis was conducted to identify the factors for achieving full immunization among children aged between 12 and 35 months in a subpopulation of the nationwide cross-sectional study conducted in June 2019 in Lao PDR. This study aimed to estimate population-based immunity against measles, rubella, and other diseases. Therefore, we set the expected measles and rubella immunoglobulin G (IgG) seroprevalence at 60% for 1–2-year-olds and 90% for children 5 years and older, level of confidence at 95%, a margin of error at 0.05, design effect at 1.6, and response rate at 99%, based on a previous study in Lao PDR [3]. The estimated sample size was 416 and 312 participants for 1–2 years old and children 5 years and older, respectively. We used a probability proportional to size sampling based on the multistage cluster method. First, 26 districts were randomly selected as primary sampling units from all districts in Lao PDR. Next, two villages were randomly selected as secondary sampling units from each district, resulting in 52 villages (Fig. 1). Subsequently, eight pairs of children and their caretakers were randomly selected after listing all residents in each village. In this study, we targeted a subpopulation of these 1–2-year-olds and their caregivers.

### Data collection

A survey team comprising two surveyors and one supervisor conducted face-to-face interviews and collected demographic information, immunization coverage, and other relevant information using structured questionnaires. Before each interview, written consent was obtained from the children’s caretakers. In addition, each child’s immunization card or mother and child health handbook were checked against the recommended EPI immunization schedule. We excluded immunization history from the caretaker’s memory because it is unreliable. Table 1 shows the recommended EPI immunization in Lao PDR during data collection. Japanese encephalitis vaccine (JEV) and rubella-containing vaccines were excluded from the full immunization assessment because they were introduced into routine immunization. However, the program is being expanded in a phase-based manner [9]. All collected data were double-entered and cleaned using a Microsoft Excel 2017 spreadsheet.

**Table 1:** Routine childhood immunization and recommended age in Lao PDR

Vaccine	Age
Hepatitis B	Birth
BCG^*1^	Birth
DTP-Hib-HepB^*2^	6, 10, and 14 weeks
OPV^*3^	6, 10, and 14 weeks
IPV^*4^	14 weeks
PCV^*5^	6, 10, and 14 weeks
JEV^*6^	9 months
Measles/rubella	9 and 12–18 months

^*1^ BCG, Bacillus Calmette-Guérin vaccine; ^*2^ DTP-Hib-Hep, diphtheria, tetanus, pertussis, Haemophilus Influenza, and hepatitis B vaccine; ^*3^ OPV, oral polio vaccine; ^*4^ IPV, inactivated polio vaccine; ^*5^ PCV, pneumococcal conjugate vaccine; ^*6^ JEV, Japanese encephalitis vaccine

### Definition of study variables

In this study, the dependent variable was immunization coverage obtained from immunization records. The immunization coverage was categorized into two groups: “fully immunized” children who received a birth dose of the Bacillus Calmette and Guérin vaccine (BCG) and Hep B; one dose of the measles-containing vaccine; and three doses of the pentavalent vaccine containing diphtheria, tetanus, pertussis, hepatitis B and haemophilus influenza (DPT-Hib-HepB), Polio vaccine, and pneumococcal conjugate vaccine (PCV) and “partially immunized” children who missed any vaccine dose. Immunization coverage was calculated as the ratio of “fully immunized” to the total. In addition, we compared demographic characteristics, health service utilization, and source of information factors in children as independent variables between the fully immunized and partially immunized groups.

Missing vaccines were also assessed based on the vaccine history to identify the gap between partial and full immunization.

### Data entry and statistical analysis

Somers’ Delta was used for continuous variables with clustered non-normal distribution to assess the relationship between immunization coverage and determinants relevant to immunization coverage. Crude odds ratios (ORs) and 95% confidence intervals (CIs) were calculated using logistic regression. Multivariate logistic regression analysis was performed for all significant factors with cluster-robust standard errors. Due to the high multicollinearity among the variables, the variable “immunization in outreach in the village” was excluded from the final multivariate logistic regression model. Furthermore, the backward stepwise selection was applied with the elimination of variables with a significance level of 0.05 from the full model. Statistical significance was set at *P* < 0.05. Statistical analyses were conducted using STATA version 16 (Stata Corp., College Station, TX, USA).

### Ethical considerations

This survey was reviewed and approved by the ethical committee of the Ministry of Health, Lao PDR (06/NECHR) and the Institutional Review Board of the National Center for Global Health and Medicine, Japan (NCGM-G-003038-00). The study described was conducted following the Declaration of Helsinki for experiments involving humans. In addition, the Ministry of Health and provincial and district government authorities arranged access to the selected households. Informed consent was obtained from all adults and children’s caretakers. After the trained surveyors explained the objectives and methodology of the study, consent was obtained from each participant. The selected children and their caretakers were given the option not to participate.

## Results

### Study profile

Data from 256 pairs of children aged 12–35 months and their caretakers were analyzed. We targeted 416 pairs of children and their caretakers to participate; 160 pairs were deemed ineligible for analysis for the following reasons: the number of children in the village was less than the planned 8 (a total of 15 in five villages); 29 pairs did not participate; 13 did not meet the age criteria (< 12 or > 35 months old); 5 participated outside the protocol; 98 pairs had no immunization records, including immunization cards or mother and child health handbooks.

Based on the reported information and verification by immunization records, 173 (67.6%) and 83 (32.4%) children were fully and partially immunized, respectively. No significant differences were observed in the demographic characteristics between the two groups of children (Table 2).

**Table 2:** Family characteristic-related and health service utilization factors of childhood vaccination status

	Fully immunized	Partially immunized	Bivariate analysis			Multivariable analysis		
			Crude odds ratio	95% CI^**a**^	***p***-value	Adjusted odds ratio	95% CI^**a**^	***p***-value
**Number**	**173**	**83**						
	67.6 (61.5–73.3) %	33.4 (26.7–38.5) %						
Sex of children								
Boy	87 (66.4%)	44 (33.6%)	0.91	0.59–1.39	0.66			
Girl	85 (68.5%)	39 (31.5%)	References					
Median of maternal age (years) (IQR^b^)	28 (24–33)	29 (25–33)			0.82			
Maternal ethnicity								
Laolum	110 (69.2%)	49 (30.8%)	1.23	0.57–2.64	0.59			
Non-Laolum	62 (64.6%)	34 (35.4%)	References					
Maternal occupation								
Farmer	125 (64.1%)	70 (35.9%)	0.49	0.20–1.21	0.12			
Not farmer	47 (78.3%)	13 (21.7%)	References					
Maternal education								
Primary school	82 (68.3%)	38 (31.7%)	1.05	0.54–2.05	0.88			
More than primary school	90 (67.2%)	44 (32.8%)	References					
Median of maternal age (years) (IQR^b^)	31 (27–37)	31 (27–37)			0.66			
Paternal ethnicity								
Laolum	110 (70.5%)	46 (29.5%)	1.36	0.62–2.95	0.44			
Non-Laolum	60 (63.8%)	34 (36.2%)	References					
Paternal occupation								
Farmer	109 (64.1%)	61 (35.9%)	0.56	0.26–1.19	0.13			
Not farmer	61 (76.3%)	19 (23.7%)	References					
Paternal education								
Primary school	54 (65.1%)	29 (34.9%)	0.82	0.49–1.36	0.44			
More than primary school	116 (69.5%)	51 (30.5%)	References					
Median number of family members (IQR^b^)	6 (4–7)	6 (4–8)			0.19			
Median number of children (< 15 years old)^c^ (IQR^b^)	2 (2–3)	2 (2–3)			0.02^*^			
House location								
Fixed	65 (84.4%)	12 (15.6%)	3.61	1.64–7.95	0.001^*^			
Others	105 (60%)	70 (40%)	References					
Median time taken to reach the nearest health facilities (minutes)^d^ (IQR^b^)	20 (10–30)	20 (10–30)			0.18			
Within 15 minutes	85 (74.6%)	29 (25.4%)	1.80	0.83–3.90	0.14			
More than 15 minutes	88 (62.0%)	54 (38.0%)	References					
Birthplace of children								
Hospital + Health facility	157 (78.1%)	44 (21.9%)	8.70	5.30–14.28	< 0.0001^*^	9.75	5.72–16.62	< 0.001^*^
Others	16 (29.1%)	39 (70.9%)	References			References		
Place to receive vaccination								
Hospital + Health facility	136 (73.9%)	48 (26.1%)	2.75	1.14–6.68	0.03^*^			
Others	36 (50.7%)	35 (49.3%)	References					
Received vaccination in outreach in the village								
Yes	77 (59.2%)	53 (40.8%)	0.46	0.22–0.94	0.03^*^			
No	95 (76%)	30 (24%)	References					

^a^ *CI* confidence interval

^b^ *IQR* interquartile range

^c^ The mean number of children less than 15 years old was 2.3 with a standard deviation of 1.2 in the fully immunized group and 2.7 with a standard deviation of 1.4 in the partially immunized group

^d^ The mean time taken to reach the nearest health facilities was 26.9 minutes with a standard deviation of 25.4 in the fully immunized group and 33.9 minutes with a standard deviation of 38.6 in the partially immunized group

^*^ *p* < 0.05 was considered statistically significant

### Factors associated with immunization coverage

The fully immunized group had fewer children < 15 years (2.3 ± 1.2 vs. 2.7 ± 1.4, *p*-value: 0.02), and residence on a fixed site had a significant association with full immunization compared to others, such as mobile (OR: 3.61, 95% CI: 1.64–7.95, *p*-value: 0.001) (Table 2).

Among health service utilization factors, childbirth and immunization at hospitals or health facilities (OR: 8.70, 95% CI: 5.30–14.28, *p* < 0.0001) and (OR: 2.75, 95% CI: 1.14–6.68, *p*-value: 0.03), respectively, were positively associated, and immunization in outreach in the village (OR: 0.46, 95% CI: 0.22–0.94, *p*-value: 0.03) was negatively associated with full immunization coverage (Table 2). No significant differences were observed in the sources of information for immunization and immunization dates (Table 3).

**Table 3:** Source of information on vaccination and vaccination date

	Fully immunized	Partially immunized	Bivariate analysis		
			Crude odds ratio	95% CI^**a**^	p-value
Source of information on vaccination					
From medical staff					
Yes	144 (68.6%)	66 (31.4%)	1.29	0.57-2.94	0.54
No	27 (62.3%)	16 (37.7%)	References		
From village health volunteer					
Yes	95 (63.3%)	55 (36.7%)	0.68	0.37-1.25	0.22
No	71 (71.7%)	28 (28.3%)	References		
From information written on the vaccination card					
Yes	64 (68.1%)	30 (31.9%)	1.11	0.56-2.19	0.77
No	100 (65.8%)	52 (34.2%)	References		
From family member					
Yes	25 (67.6%)	12 (32.4%)	1.05	0.40-2.74	0.92
No	139 (66.5%)	70 (33.5%)	References		
From friends					
Yes	13 (68.4%)	6 (31.6%)	1.07	0.36-3.19	0.90
No	150 (67.0%)	74 (33.0%)	References		
From radio/TV					
Yes	20 (57.1%)	15 (42.9%)	0.60	0.29-1.27	0.18
No	144 (68.9%)	65 (31.1%)	References		
From poster					
Yes	11 (64.7%)	6 (35.3%)	0.89	0.30-2.59	0.83
No	153 (67.4%)	74 (32.6%)	References		
From village head					
Yes	84 (61.8%)	52 (38.2%)	0.59	0.33-1.07	0.08
No	82 (73.2%)	30 (26.8%)	References		
From women’s union					
Yes	19 (52.8%)	17 (47.2%)	0.50	0.25-0.996	<0.05
No	146 (69.1%)	65 (30.9%)	References		
Source of information on vaccination date					
From medical staff					
Yes	114 (70.4%)	48 (29.6%)	1.35	0.76-2.42	0.31
No	58 (63.7%)	33 (36.3%)	References		
From village health volunteer					
Yes	98 (63.2%)	57 (36.8%)	0.70	0.37-1.31	0.26
No	64 (71.1%)	26 (28.9%)	References		
From the information written on the vaccination card					
Yes	65 (71.4%)	26 (28.6%)	1.42	0.70-2.88	0.34
No	97 (63.8%)	55 (36.2%)	References		
From family member					
Yes	26 (66.7%)	13 (33.3%)	1.01	0.41-2.49	0.98
No	136 (66.3%)	69 (33.7%)	References		
From friends					
Yes	9 (64.3%)	5 (35.7%)	0.89	0.27-2.96	0.85
No	153 (66.8%)	76 (33.2%)	References		
From radio/TV					
Yes	5 (55.6%)	4 (44.4%)	0.61	0.14-2.69	0.52
No	157 (67.1%)	77 (32.9%)	References		
Froom poster					
Yes	106 (66.7%)	53 (33.3%)	1.05	0.57-1.94	0.87
No	57 (65.5%)	30 (34.5%)	References		
From village head					
Yes	18 (54.5%)	15 (45.5%)	0.55	0.24-1.29	0.17
No	145 (68.4%)	67 (31.6%)	References		
From woman’s union					
Yes	41 (70.7%)	17 (29.3%)	1.29	0.54-3.04	0.57
No	120 (65.2%)	64 (34.8%)	References		
From megaphone					
Yes	13 (54.2%)	11 (45.8%)	0.56	0.17-1.86	0.34
No	148 (67.9%)	70 (32.1%)	References		
From an official letter from the district governor					
Yes	4 (80%)	1 (20%)	2.03	0.22-18.96	0.54
No	158 (66.4%)	80 (33.6%)	References		

^a^ *CI* confidence interval

The multivariate logistic regression model revealed that childbirth at a hospital or health facility (AOR: 9.75, 95% CI: 5.72–16.62, *p* < 0.001) was significantly associated with complete immunization coverage (Table 2).

### Missing vaccines in the partially immunized group

Of the 83 children in the partially immunized group, 46 (55.4%) did not receive the Hep B at birth, 34 (41.0%) had not completed three doses of PCV, and 27 (32.5%) had not received their first measles vaccination. In contrast, 70 (84.3%), 67 (80.7%), and almost all (82, 98.8%) had received three doses of the pentavalent vaccine, completed three doses of polio vaccination, and received the BCG vaccine, respectively.

## Discussion

To the best of our knowledge, this is the first study to examine the immunization coverage nationwide among children aged 12–35 months using multistage cluster sampling and investigate the determinants of full immunization with a birth dose of Hep B after introducing recently implemented efforts to improve vaccination.

Our study indicated that childbirth at a hospital or health facility was significantly associated with complete immunization. This is related to access to health services, which is similar to previous studies in Lao PDR [10–14]. Lao PDR is ethnically diverse, including 49 ethnic groups, most of whom live in rural and remote mountainous areas, with limited communication, transport, and social service provision [15]. Therefore, access to health services remains a significant barrier to immunization coverage. In addition, comprehensive and appropriate information dissemination is important for immunization against EPI-covered diseases [16]. The utilization of micro-planning for immunization sessions and the activities of mobile teams have also been suggested to be successful in providing routine immunization [5]. Therefore, adapting these activities to local conditions is necessary to improve immunization coverage.

However, the proportion of children (173 children, 67.6%) who were fully immunized was lower than the national target of 90%. The definition and rates of full immunization varied among studies in Southeast Asian (SEA) countries. Previous studies have reported complete immunization rates of 59.0–80.8%, 79.3–86.4%, and 55.4% in Lao PDR [14, 16, 17], Malaysia [18, 19], and Myanmar [20], respectively. Several factors are associated with immunization coverage in SEA countries. Sociodemographic characteristics affecting complete immunization are the number of children in the family, child’s age, child’s ethnicity, mother’s age, mother’s ethnicity, mother’s religion, mother’s education, mother’s occupation, father’s education, father’s occupation, zone of residence, travel time to health facilities, and willingness to pay for immunization [14, 16–22]. Health system and service utilization factors, including mother’s antenatal care attendance, tetanus vaccination during pregnancy, and delayed immunization schedule, are also associated with immunization coverage [18, 20]. Here, the univariate analysis showed that non-residency in a fixed house and a greater number of children were associated with partial immunization. However, several countermeasures have been explored to improve vaccine coverage. In low- and middle-income countries, education may be more effective than incentives to increase vaccination [23]. Similarly, it has been suggested that soft skills, including communication by community outreach teams regarding immunization activities in Lao PDR, also help residents’ vaccine acceptance [13]. Therefore, the effective utilization of the health system and services should also be considered to achieve the national target of full immunization.

Here, full immunization was defined as having received eight doses of vaccines included in the WHO definition of full immunization (one dose of BCG vaccine, three doses of the polio vaccine, three doses of *diphtheria-tetanus-pertussis* vaccine, and one dose of measles-containing vaccine) [24], plus a birth dose of Hep B and three doses of PCVs considering the vaccine introduction situation in Lao PDR [8]. In addition, the following vaccines were identified as factors contributing to partial immunization: a birth dose of Hep B, three doses of PCVs, and one dose of measles-containing vaccine, and non-coverage rates for these vaccines were similar to those previously reported [8, 25]. Furthermore, the immunization coverage we identified was similar to that previously reported (59.0–80.1%) [14, 16]; however, differences in the definition of full immunization may have had an impact.

The strength of our study is that we surveyed individuals with documented immunization records on a nationwide scale and selected participants using random sampling, which has the advantage of accurately assessing the situation throughout the country. However, this study had some limitations. First, the survey has the advantage of being able to assess immunization status on a national scale; however, groups with varying immunization statuses, including ethnic minorities, may be elusive. For example, in Lao PDR, a measles outbreak was reported in 2019 in an ethnic minority group with low immunization coverage. Therefore, national policies should recognize this heterogeneity. Second, this study analyzed subpopulations of the nationwide measles and rubella seroepidemiological survey. Therefore, the study design and sample size followed this survey, which assessed measles and rubella immunity nationwide. Thirdly, against the targeted 416 pairs, 256 pairs were finally included in the analysis and 160 pairs were excluded from the analysis. Among them, 98 pairs without vaccination records were excluded from the analysis. Therefore, although the current situation requires immunization records, including those used in this study, the results may have selection bias [13]. However, the issues raised in our study warrant further investigation into subgroups of ethnic minorities using mobile device apps for immunization records.

## Conclusions

Our study elucidated that the immunization status among children aged between 12 and 35 months in Lao PDR is satisfactory in improving access to healthcare by strengthening communication with residents regarding health service utilization, and expanding mobile outreach services may play a pivotal role in this endeavor. Further research is warranted to evaluate efforts to increase immunization coverage and target populations with limited access to healthcare.

## Data Availability

The data supporting the present study’s findings are available from the corresponding author, Yasunori Ichimura, on reasonable request.

## Abbreviations

Lao PDR: Lao People’s Democratic Republic
DTP-Hib-HepB: Diphtheria, Tetanus, Pertussis, Hepatitis B and Haemophilus Influenza
PCV: Pneumococcal conjugate vaccine
AOR: Adjusted odds ratio
CI: Confidence interval
EPI: Extended program on immunization
WHO: World Health Organization
UNICEF: United Nations Children’s Fund
SIAs: Supplementary immunization activities
NIP: National Immunization Program
MCV: Measles-containing vaccine
JEV: Japanese Encephalitis Vaccine
BCG: Bacillus Calmette and Guérin vaccine
Hep B: Hepatitis B vaccine
SEA: Southeast Asian
